# *In silico* interrogation of the miRNAome of infected hematopoietic cells to predict processes important for human cytomegalovirus latent infection

**DOI:** 10.1016/j.jbc.2023.104727

**Published:** 2023-04-19

**Authors:** M.J. Murray, E. Bradley, Y. Ng, O. Thomas, K. Patel, C. Angus, C. Atkinson, M.B. Reeves

**Affiliations:** Institute of Immunity & Transplantation, Division of Infection & Immunity, Royal Free Campus, UCL, London, United Kingdom

**Keywords:** human cytomegalovirus, miRNA, cell signalling, viral entry, latency

## Abstract

Human cytomegalovirus (HCMV) latency in CD34+ progenitor cells is the outcome of a complex and continued interaction of virus and host that is initiated during very early stages of infection and reflects pro- and anti-viral activity. We hypothesized that a key event during early infection could involve changes to host miRNAs, allowing for rapid modulation of the host proteome. Here, we identify 72 significantly upregulated miRNAs and three that were downregulated by 6hpi of infection of CD34+ cells which were then subject to multiple *in silico* analyses to identify potential genes and pathways important for viral infection. The analyses focused on the upregulated miRNAs and were used to predict potential gene hubs or common mRNA targets of multiple miRNAs. Constitutive deletion of one target, the transcriptional regulator JDP2, resulted in a defect in latent infection of myeloid cells; interestingly, transient knockdown in differentiated dendritic cells resulted in increased viral lytic IE gene expression, arguing for subtle differences in the role of JDP2 during latency establishment and reactivation of HCMV. Finally, *in silico* predictions identified clusters of genes with related functions (such as calcium signaling, ubiquitination, and chromatin modification), suggesting potential importance in latency and reactivation. Consistent with this hypothesis, we demonstrate that viral IE gene expression is sensitive to calcium channel inhibition in reactivating dendritic cells. In conclusion, we demonstrate HCMV alters the miRNAome rapidly upon infection and that *in silico* interrogation of these changes reveals new insight into mechanisms controlling viral gene expression during HCMV latency and, intriguingly, reactivation.

The interaction between viruses and hosts is multifaceted and complex. These interactions represent both proviral and antiviral mechanisms that dictate the final outcome of infection. Here we study this concept through an investigation of the interaction of human cytomegalovirus (HCMV) with CD34+ hematopoietic progenitor cells during the very early stages of cell infection. Specifically, the changes virus binding and entry at the plasma membrane can induce are investigated to understand the importance of these interactions for the establishment of viral latency in CD34+ progenitor cells.

MicroRNAs (miRNAs) are a family of small RNA species that predominantly operate to regulate protein expression through the destruction of mRNA transcripts or *via* the inhibition of translation (1, 2). The specificity of miRNAs requires sequence-specific detection of nucleotide sequences within the mRNA to provide targeting. Importantly, this sequence specificity allows the prediction of mRNA targets using bioinformatics approaches which can then be validated experimentally. Pertinently, it has been demonstrated that HCMV dramatically alters the host miRNAome during lytic infection and during long-term latent infection of CD34+ cells (2, 3). Furthermore, the importance of miRNAs for HCMV infection is supported by the identification of several HCMV-encoded miRNAs that further manipulate the host cell environment (4, 5). It is unlikely that changes in the miRNAome impart gross changes in the proteome but rather acts as a rheostat to fine-tune the proteome optimizing the host environment for viral infection.

The contribution of host and virally encoded miRNAs to the regulation of herpesvirus latency has been well documented. For example, herpes simplex virus-1 (HSV-1) encodes several miRNAs that promote the destruction of transcripts that drive lytic infection (6, 7). An elegant study demonstrated that these effects are augmented by elevated expression of the host miRNA miR-138 in neuronal cells—a key site of HSV latency (8). One target of miR-138 is the HSV ICP0 mRNA, again serving to promote the silencing of lytic gene expression. Subsequently, a similar model has been proposed for HCMV whereby CD34+ hematopoietic cells express high levels of miR-200 which targets the UL122 transcript for destruction—a functional equivalent of ICP0 in HSV-1 (9). Once again, HCMV itself encodes for an additional miRNA (miR-UL112.1) which limits translation from the UL123 mRNA—an activity also consistent with latency (10, 11). Finally, the concept of host miRNAs that target and limit viral gene expression being expressed in cells that restrict lytic infection has also been shown in HIV and thus resonates across diverse virus families (12, 13, 14).

Importantly, these miRNA functions are not working in isolation and, instead, likely serve to augment other mechanisms that regulate viral gene expression important for the establishment of viral latency (*e.g.* chromatin-mediated silencing, cell signaling pathways, and immune evasion). For example, HCMV inhibits the expression of miR-92a in long-term latently infected cells (3, 15) which serves to promote the up-regulation of the cellular transcription factor GATA-2 and the activation of immune-suppressive IL-10 (16). Indeed, latently infected cells have been shown to induce IL-10 and TGF-β which protects them from elimination by immune cells (17). The targeting of GATA-2 also promotes latent viral gene expression as many promoters of viral genes expressed during latency contain GATA-2 binding sites (18, 19). In addition, virally encoded miRNAs target transforming growth factor β (TGF-ß) in a complex way. The removal of NAB1 by miR-US5-2 promotes TGF-ß release which likely contributes to the immune-suppressive phenotype observed in latently infected cells as well as to promote myelosuppression (20). Also, given the essential role TGF-ß plays in dendritic cell commitment, these interactions may be important for the persistence of HCMV in the myeloid lineage. Finally, HCMV encodes additional miRNAs (miR-US25–1 and UL148D) which function to limit TGF-ß–induced cell death and activin-induced IL-6 immune inflammation (21, 22).

What these examples illustrate is that the targeting of host cellular functions by viruses is usually multifaceted and is more complex than just activation of inhibition. Instead, it is likely that manipulation of the host miRNAome and proteome alongside expression of viral effector functions provides a strategy for a pathogen to utilize the aspects that provide benefit while disabling aspects less beneficial. Furthermore, it is highly plausible the virus initiates some changes during the early stages of infection which are sustained long term through additional functions. Furthermore, even short-term changes that occur during the initial stages of viral infection have the potential to have a long-term impact—for example, any event that limits the establishment of viral latency will manifest as a defect in reactivation (since reactivation is inherently a “readout” of latency).

Herein we present data from infected CD34+ cells that demonstrate discrete changes in the miRNAome occur within 6hpi with HCMV. Validation of targets by qPCR reveals that changes in the miRNAome are not observed in infected fibroblasts arguing that these represent myeloid-specific changes. Furthermore, analyses of specific miRNAs suggest that these changes are transient arguing for the changes being a result of the initial interactions of HCMV with the myeloid cells. Using algorithms based on experimental and bioinformatic data we predict potential protein targets that will likely be downregulated by a panel of upregulated miRNAs which we hypothesized to reveal the biggest effects due to concerted activity against a pathway. We then used cluster analysis to identify host cell functions that could potentially be targets for manipulation which can be tested experimentally. In doing so, we provide a miRNA dataset resource of potential interest to colleagues in the field alongside a report on the results of our *in silico* approaches that extrapolate from the dataset to predict potential host functions that are targets for HCMV infection. Finally, we demonstrate that using this approach identified a novel role for calcium ion channel signaling in both HCMV latency and, intriguingly, reactivation.

## Results and discussion

### HCMV infection promotes the upregulation of a subset of host-encoded miRNAs in CD34+ cells

**Figure 1:**
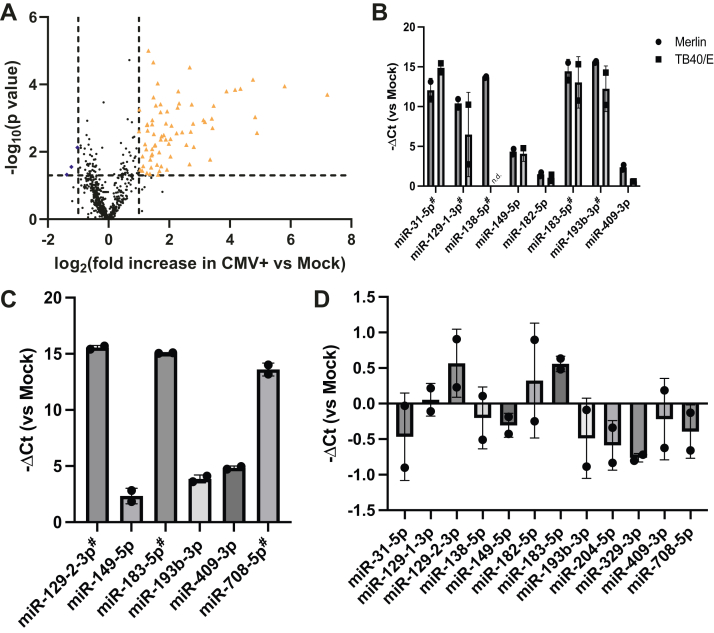
**HCMV infection of myeloid cells triggers induction of host miRNAs.***A*, CD34+ cells were infected at MOI = 5 with HCMV strain Merlin (n = 3). Media was replaced 3 h post-infection, and 6 hours post-infection; miRNA was extracted and assessed by qPCR array for 1055 miRNAs. *Orange triangles* represent miRNAs upregulated >2-fold with *P* < 0.05, *blue diamonds* represent miRNAs downregulated >2-fold with *P* < 0.05, *P*-values calculated by student’s *t* test. *B*, Primary CD34+ cells were infected at MOI = 5 with Merlin or TB40/E (n = 2 independent replicates). Six hours post-infection, miRNA was extracted and assessed by specific qRT-PCR, with expression displayed as −ΔCt relative to expression in uninfected cells or a technical cutoff. *C* and *D*, Primary CD14+ monocytes (*C*) or primary fibroblasts (*D*) were infected at MOI = 5 with Merlin (n = 2 independent replicates). Six hours post-infection, miRNA was extracted and assessed by specific qRT-PCR, with expression displayed as −ΔCt relative to expression in uninfected cells or a technical cutoff if undetected in uninfected cells (indicated by #). All summary values are displayed as mean±SD. HCMV, Human cytomegalovirus; miRNAs, microRNA.

To begin to study the impact of viral binding and entry on miRNA expression total RNA was isolated at 6hpi from CD34+ cells infected at an multiplicity of infection (MOI) = 5 and subjected to an analysis of differential expression of 1055 miRNAs. Using this approach, we identified that 72 miRNAs were upregulated at least 2-fold (with *p* < 0.05) in the population of infected CD34+ cells compared to mock cells, while only three miRNAs were significantly downregulated (Fig. 1*A* and Table S1). Thus, most of the changes observed in the miRNAome upon HCMV infection represented an upregulation of miRNA expression.

To validate the data generated by the screen, CD34+ cells were infected (MOI = 5) with either Merlin (as in the original screen) or with a second strain of HCMV, TB40/E. At 6hpi, miRNAs were extracted, subjected to first-strand cDNA synthesis, and quantified by qPCR using primers generated independently of those used in the screen, focusing on those miRNAs shown to be most highly upregulated by the screen (Fig. 1*B*). The data demonstrate strain-independent induction of five of the most highly upregulated miRNAs, in comparison to uninfected cells. It should be noted that the extremely high level of apparent induction for some of these miRNAs is due to the complete absence of detection of those transcripts in the uninfected conditions, therefore generating extremely high “relative” expressions (Ct values available in Table S2). Notably, while limited by cell availability, it was possible to demonstrate that these changes were relatively transient in nature, with the increases in miRNA expression reduced or absent by 24hpi (Fig. S1*A*). Of note, infection with UV-inactivated HCMV was observed to dramatically limit the induction of all miRNA expression (Fig. S1*B*) suggesting a requirement for viral transcription. In part, this was consistent with data from experiments whereby HCMV infection was performed in the presence of viral entry inhibitor EIPA (Fig. S1*C*) where we again observed that the upregulation of some, but not all, of the subset of miRNAs analyzed by qPCR was lost by EIPA treatment (Fig. S1*D*). Surprisingly, however, we clearly noted a subset of the miRNAs were elevated in infected cells that had been pretreated with EIPA in comparison to solvent control which may suggest they are activated by virus binding which potentially could be prolonged in the EIPA-treated cells in which virus internalization is reduced. Importantly, the comparison of the EIPA and UV inactivation data (where a global shutdown of miRNA induction was observed) could suggest that the UV inactivation of HCMV may have induced additional changes to the virion beyond DNA damage that change a key binding interaction responsible. Taken together, these data suggest the observed changes in miRNA expression observed at 6hpi are multifactorial and triggered by both virus binding and events occurring post-entry (including, potentially, transcription).

To probe how specific this response reported in CD34+ cells was, miRNA induction at 6hpi in response to HCMV infection in CD14+ monocytes was analyzed. CD14+ monocytes are a commonly used cell type to model events important for viral latency. In the case of CD14+ monocytes (Fig. 1*C*), we were again able to detect the upregulation of multiple miRNAs that were also highly upregulated in the original screen. In contrast, an analysis of the same miRNAs in infected fibroblasts revealed only minor changes (∼2-fold increase/decrease) in response to infection (Fig. 1*D*). These minor changes reflect the fact that all 12 of the most highly upregulated miRNAs identified in the original screen are constitutively expressed within primary fibroblasts, but only a fraction are constitutively expressed in either CD34+ cells or CD14+ monocytes.

### *In silico* prediction of pathways involved in HCMV latent infection

The overarching rationale for studying changes in the miRNAome during the early stages of HCMV infection of CD34+ cells was that they may reveal target pathways and/or proteins that could be important for the establishment of HCMV latency. Thus, our first approach was to investigate whether there was an enrichment for miRNAs that have been demonstrated to target specific cellular pathways and thus suggest a potential role in HCMV infection.

**Figure 2:**
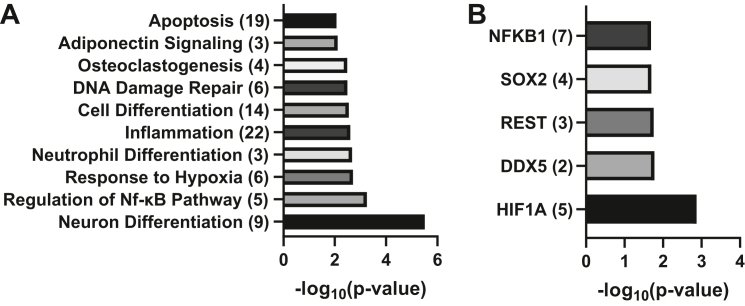
**Processes associated with inflammation and cellular differentiation are enriched within the upregulated miRNAs.** The list of upregulated miRNAs was uploaded to the TAM2.0 webserver, with the list of interpretable miRNAs as a background. The top 10 most statistically enriched functional annotations (*A*) and top five most enriched transcription factor annotations (*B*) are displayed. Value in parentheses indicates the number of miRNAs associated with the indicated function/transcription factor. miRNAs, microRNA.

Given that only three miRNAs were downregulated the study focused the pathway analyses on the upregulated miRNAs. To do this, pathway enrichment analysis was performed directly upon the list of identified upregulated miRNAs. The list of 72 miRNAs upregulated at least 2-fold was submitted to the TAM 2.0 webtool (23, 24), with the list of all quantifiable miRNAs from the original analysis used as baseline (Table S3). The most statistically enriched functions (Fig. 2*A*) included inflammation, response to hypoxia, regulation of NF-κB pathway, DNA damage repair, and apoptosis, all logical processes to be regulated in response to virus infection and could reflect pro and anti-viral events. Furthermore, we also observed pathways associated with cellular differentiation were also highly enriched. Hypothetically, this could represent an immediate attempt by the virus to regulate the differentiation state of multipotent CD34+ cells and to direct them down a particular differentiation pathway. The enrichment of these pathways is likely explained by the identification of pathways associated with key transcriptional regulators that are predicted to be targeted by multiple miRNAs seen to be upregulated (Fig. 2*B*). For instance, NF-kB activity is intimately associated with inflammation and cell death, whereas the epigenetic modifier REST complex plays a key role in the regulation of a number of host genes important for neuronal differentiation. Hypoxia-inducible factor 1α is a major component of the hypoxic response. The importance of these targets underpins the enrichment of the pathways identified (Fig. 2*A*).

### *In silico* prediction of protein targets of upregulated miRNAs

Although interesting, we considered whether the pathway analysis approach was inherently weighted toward certain well-characterized genes considered to be critical in specific pathways and thus we may miss other interesting, less well understood, hits. Consequently, the next approach first cataloged the potential protein targets of the identified upregulated miRNAs. To do this, the miRNA list was submitted to the Mienturnet web tool (25). This allows for the identification of potential targets using two different databases: TargetScan (26), which employs computational predictions to identify potential mRNA binding sites, and mRiTarBase (27), which collates data based on experimentally validated miRNA-target interactions. We surmised that combining data from these two different approaches provided the best opportunity to generate a comprehensive panel of targets that could be regulated by miRNAs during the early stages of HCMV infection of CD34+ cells.

Submission of miRNAs upregulated at least 2-fold with *P* < 0.05 with the TargetScan database resulted in the identification of 4991 proteins that were potentially targeted by at least two miRNAs. Of these, 325 had *P* < 0.05 and were targeted by between 2 and 15 miRNAs. Interrogation *via* miRTarBase resulted in the identification of 3042 proteins targeted by at least two miRNAs, with 825 having *P* < 0.05, each targeted by between 2 and 17 miRNAs. Given the large number of proteins identified, it was reasoned that the most feasible starting point for the identification of biologically relevant targets would lie within either the most confident hits from either database or within the hits that emerged from both databases. Consequently, the top 10 hits from each database were combined with the hits that were identified by both (of which there were 27) to produce a list of 46 candidate proteins (Table S4) (RIMS3 being found in both the shared list and the TargetScan list).

To test this predictive approach, the list to identify potential target proteins was examined based on previous literature. Of interest was the identification of the gene KDM1A which encodes for the lysine-specific histone demethylase 1A (LSD1). Post-translational modification of histones plays a key role in the regulation of viral gene expression, particularly during the latent lifecycle of multiple herpes viruses (28, 29, 30, 31, 32). A pharmacological inhibitor of LSD1 has previously been shown to inhibit both HSV-1 and HCMV during lytic infection and additionally inhibited HSV-1 reactivation from latency (33). Interestingly, a known regulator of LSD1, the E3 ubiquitin ligase Jade-2 (encoded by the PHF-15 gene), was also predicted to be targeted by the upregulated miRNAs. As Jade-2 activity leads to the degradation of LSD1 (34), it would seem counter-intuitive to down-regulate the expression of both proteins. However, if this was indeed the case experimentally, it could be an example of the analyzed miRNA responses representing a mixture of both pro- (Jade-2 targeting) and anti-viral (LSD1 targeting) activity.

Another gene/protein that was of potential interest was Jun dimerization protein 2 (JDP2). A major target for JDP2 mediated regulation is the AP-1 transcription factor (35, 36), binding sites for which are known to be located within the major immediate early promoter (MIEP) of HCMV (37). AP-1 binding to the MIEP has also been shown to be important for the regulation of HCMV reactivation (38), while JDP2 itself has been shown to play a role in repressing the BZLF1 promoter of Epstein-Barr virus (EBV) and consequently regulate the maintenance of EBV latency (39).

**Figure 3:**
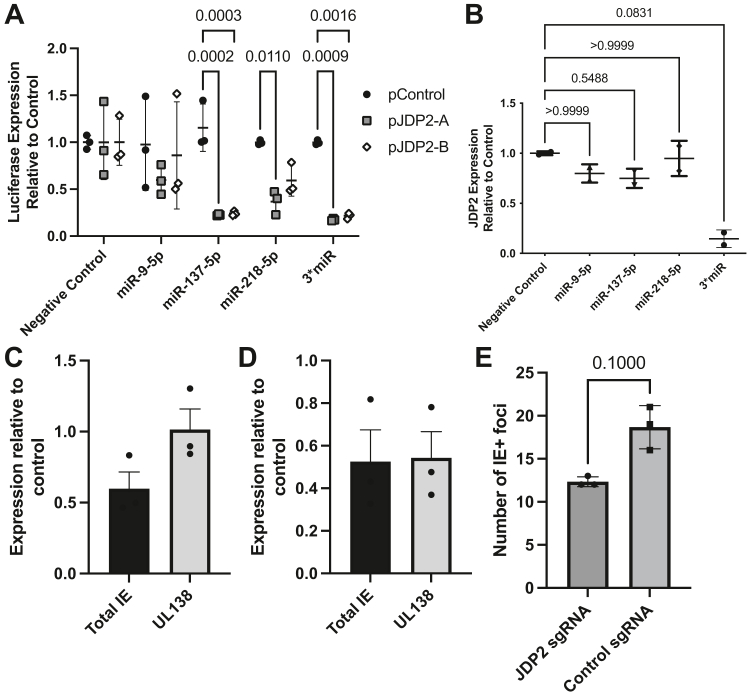
**Predicted miRNAs target JDP2 3′ UTR-containing transcripts, and JDP2 knockout reduces HCMV gene expression and reactivation in THP-1 cells.***A*, HEK-293T cells were transfected with luciferase-expressing plasmids tagged with part of the JDP2 3′ UTR (pJDP2-A and pJDP2-B) or and untagged control (pControl), along with individual miRNA mimics or a combination of 3 (3∗miR) predicted to target JDP2. Luciferase activity was detected 24 h later and expressed relative to activity in cells transfected with a negative control miRNA for each plasmid. Statistical analysis by two-way ANOVA with Tukey’s multiple comparison test (n = 3 independent replicates). *B*, HEK-293T cells were transfected with miRNA mimics as in (*A*). 24 h later, JDP2 expression was enumerated by RT-qPCR and expressed relative to expression in negative control miRNA-transfected cells (n = 2 independent replicates). Statistical comparison by one-way ANOVA with non-parametric Kruskal–Wallis test with Dunn’s multiple comparison correction. *C*–*E*, JDP2 knockout THP-1 cells were infected at MOI = 5 with HCMV strain TB40/E. RNA was extracted 1 day (*C*) or 3 days (*D*) post-infection, and viral gene transcription was analyzed by qRT-PCR. Gene expression was displayed as a relative expression in JDP2 knockout cells compared to control sgRNA-expressing cells (n = 3 independent replicates). *E*, Alternatively, at 5 days post-infection cells were differentiated with PMA, followed by overlay with fibroblasts. Cells were fixed and IE+ foci were enumerated by immunostaining and immunofluorescence, statistical comparison by non-parametric Mann–Whitney *U* test (n = 3 independent replicates). Summary data are displayed as mean ± SD throughout. HCMV, Human cytomegalovirus; miRNAs, microRNA.

Given these intriguing links between JDP2 and the potential to control viral latency, it was asked whether this predictive approach could inform new biology of HCMV infection. Thus, the first step was to test whether the upregulated miRNAs predicted to target JDP2 could indeed regulate JDP2 expression. First, the ability of miRNA mimics (miR-9-5p, miR-137-5p and miR-218-5p), either individually or as a combination of all three (3∗miR), to affect expression of a luciferase construct tagged with the JDP2 3′ UTR (split between two plasmids due to its length, pJDP2-A and pJDP2-B) was compared to an untagged control plasmid (pControl) and to a non-targeting miRNA mimic (Fig. 3*A*). Luciferase activity from pJDP2-A and pJDP2-B was highly significantly reduced by miR-137-5p and the 3∗miR combination, while pJDP2-A activity was also highly reduced by miR-218-5p, with pJDP2-B demonstrating a noticeable but not statistically significant reduction by miR-218-5p. This confirmed that our predictions could be validated *in vitro*. We further sought to validate our predictions by assessing the impact of these miRNA mimics on JDP2 expression levels in HEK-293T cells (Fig. 3*B*) and THP-1 cells (Fig. S2). RNA samples were isolated from cells 24h post-transfection with individual or a combination of miRNA mimics, and JDP2 expression assessed by qPCR. Whilst individual miRNA mimics did not exhibit much of an impact, combination treatment highly reduced JDP2 mRNA levels. This impact in the combined setting only provides further evidence that an understanding of the interaction of multiple miRNAs with their putative targets, rather than focusing on the effect of a singular miRNA on a singular transcript, is important experimentally.

Having demonstrated the miRNAs regulated JDP2 and that the rationale for the approach was to identify novel regulators of viral latency and reactivation it was next asked whether JDP2 has any role in HCMV infection. CRISPR-Cas9–mediated knockout was performed using three independent JDP2-targeting sgRNAs to generate three JDP2 knockout THP-1 (a commonly used model of latency) cell lines. These cells were then infected at MOI = 5, and RNA was harvested after 1 and 3 days. These RNA samples were interrogated by qRT-PCR for the presence of total IE gene expression, typically a marker of abortive lytic infection in these cells, and UL138, one of a subset of viral transcripts produced during both lytic and latent infection. At 1dpi (Fig. 3*C*), we observed a 40% decrease in the production of IE transcripts, but little impact on the expression of UL138, while at three dpi (Fig. 3*D*), both total IE and UL138 transcription were reduced by ∼45% compared to control cells. In parallel, a subset of the infected THP1 cells were maintained for 5 days prior to differentiation with PMA, to trigger HCMV reactivation. The differentiated cells were overlaid with HFFs, and the number of IE+ foci enumerated (Fig. 3*E*). Here, we also observed a reduction in the number of IE+ foci in the JDP2 KO co-cultures. Given that JDP2 is known to regulate an activator of the MIEP we were surprised to observe that loss of JDP2 did not promote IE gene expression. Indeed, during the early stages of infection, JDP2 KO was resulting in a loss of MIE activity (but not a comparable effect on the expression of UL138). Thus, the data suggested JDP2 KO was having a negative impact on latency and reactivation, but this was complicated by the understanding that latency and reactivation are linked—less efficient latency will manifest as less efficient reactivation. Thus, to address this potential confounder of a defect in the establishment of latency, CD14+ monocytes isolated from healthy donors were infected with HCMV to establish latency and then differentiated into immature dendritic cells (MoDCs) over 6 days. At day 4, during differentiation (so post-establishment of latency), the cells were treated with either JDP2 or a negative control siRNA (Fig. S3*A*) to deplete JDP2 mRNAs. This approach allowed interrogation of the role of JDP2 specifically during HCMV reactivation. In unstimulated MoDCs, it was observed that JDP2 depletion alone resulted in increased IE gene expression from latent HCMV. Notably, when reactivation was stimulated with IL-6 any minor phenotype associated with a reduction in JDP2 levels was lost. Thus, JDP2 has a role in silencing of IE gene expression prior to stimulation with a robust trigger of HCMV reactivation but is unlikely to have a role in controlling the switch (*e.g*. IL-6).

### *In silico* prediction of clusters important in HCMV infection

**Figure 4:**
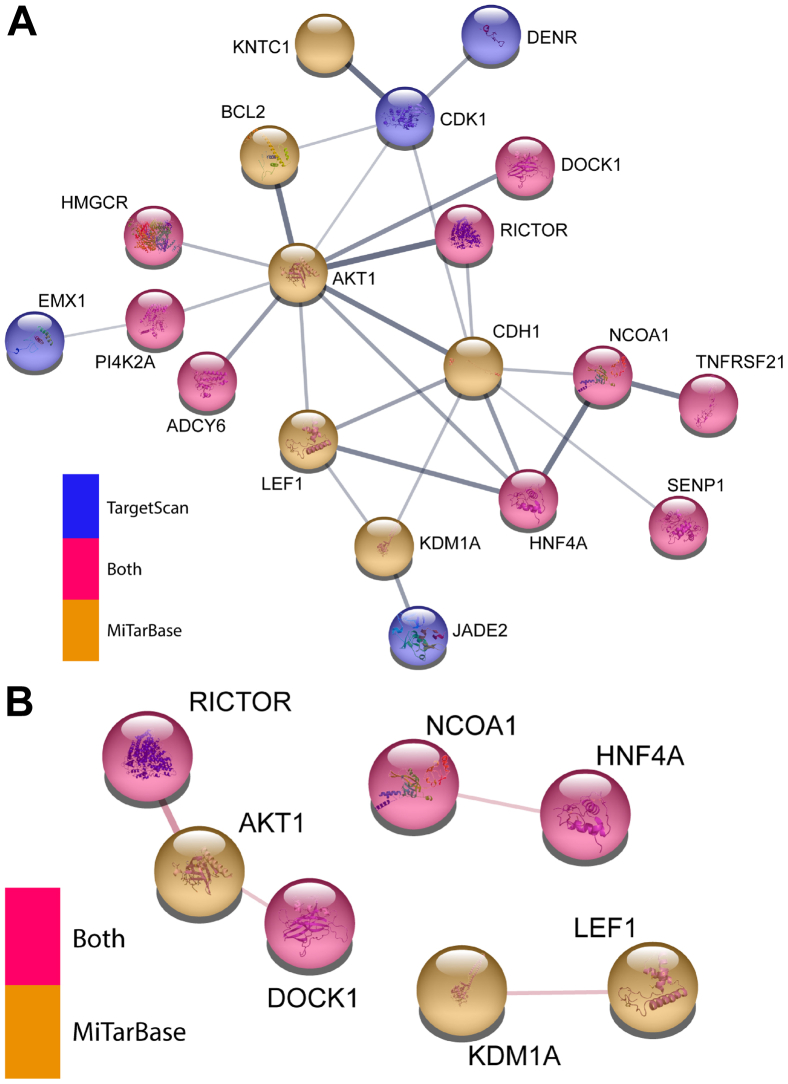
**Analysis of protein-protein interactions within the initial list of miRNA-targeted proteins reveals a single highly connected cluster.** The initial list of 46 genes was uploaded to the STRING webserver to assess predicted protein-protein interactions. Functional (*A*) and physical (*B*) interaction networks were produced. Interactions were of confidence >0.4, and only proteins predicted to interact with at least one other analyzed protein at this confidence level were displayed. Protein coloration indicates which database(s) their respective genes originated from. HCMV, Human cytomegalovirus; miRNAs, microRNA.

The COVID-19 pandemic and UK lockdown situation led us to consider more complex ways of interrogating the miRNA dataset *in silico* with a view to developing novel interpretations of the dataset from which we could generate testable hypotheses. Consequently, we supplemented the original pathway data from the miRNA analyses (Fig. 2) with interaction and clustering data based on the identified proteins (Table S4). Submission of the initial list of 46 proteins to the STRING database of protein-protein interactions (40) resulted in a group of 17 proteins thought to associate (*i.e.* contribute to a shared function, not necessarily physically interact) with one another (with at least “medium”, 0.4 on scale 0–1, confidence), with a key hub protein being AKT1 (Figs. 4*A* and S4*A*). The only other association at this level of confidence was between PI4K2A and EMX1, which was based solely on co-mentions in PubMed abstracts. Such a large single group would be hard to experimentally interrogate, particularly as it includes AKT1, BCL2, and CDK1, disruption to the functions of which would likely have a severe impact upon the status of the cell even in the absence of HCMV infection. When solely physical interactions were considered, only a very small number of interactions were reported by STRING (Figs. 4*B* and S4*B*). As a result, the outputs from Mienturnet were reanalyzed by a range of different approaches to try and identify clusters of related proteins.

### Stringent analysis of TargetScan results identifies ubiquitin-associated protein cluster

Rather than selecting common proteins and top hits from each of the databases, a more stringent criteria was applied to select proteins from a single database. For instance, we selected proteins with *P* < 0.05 that were targeted by at least five different miRNAs from the same list of protein outputs from the TargetScan database as used previously. This led to a new list of 186 proteins (Table S4), which were then again submitted to STRING to attempt to identify related groups of proteins. Given the increased number of proteins being submitted, we elected to focus initially on interactions considered to be of the “highest” confidence, as determined by STRING. This would likely require there to be experimental evidence of said interaction, allowing us to reinforce the “predicted” targets of the miRNAs with data arising from direct experimentation.

**Figure 5:**
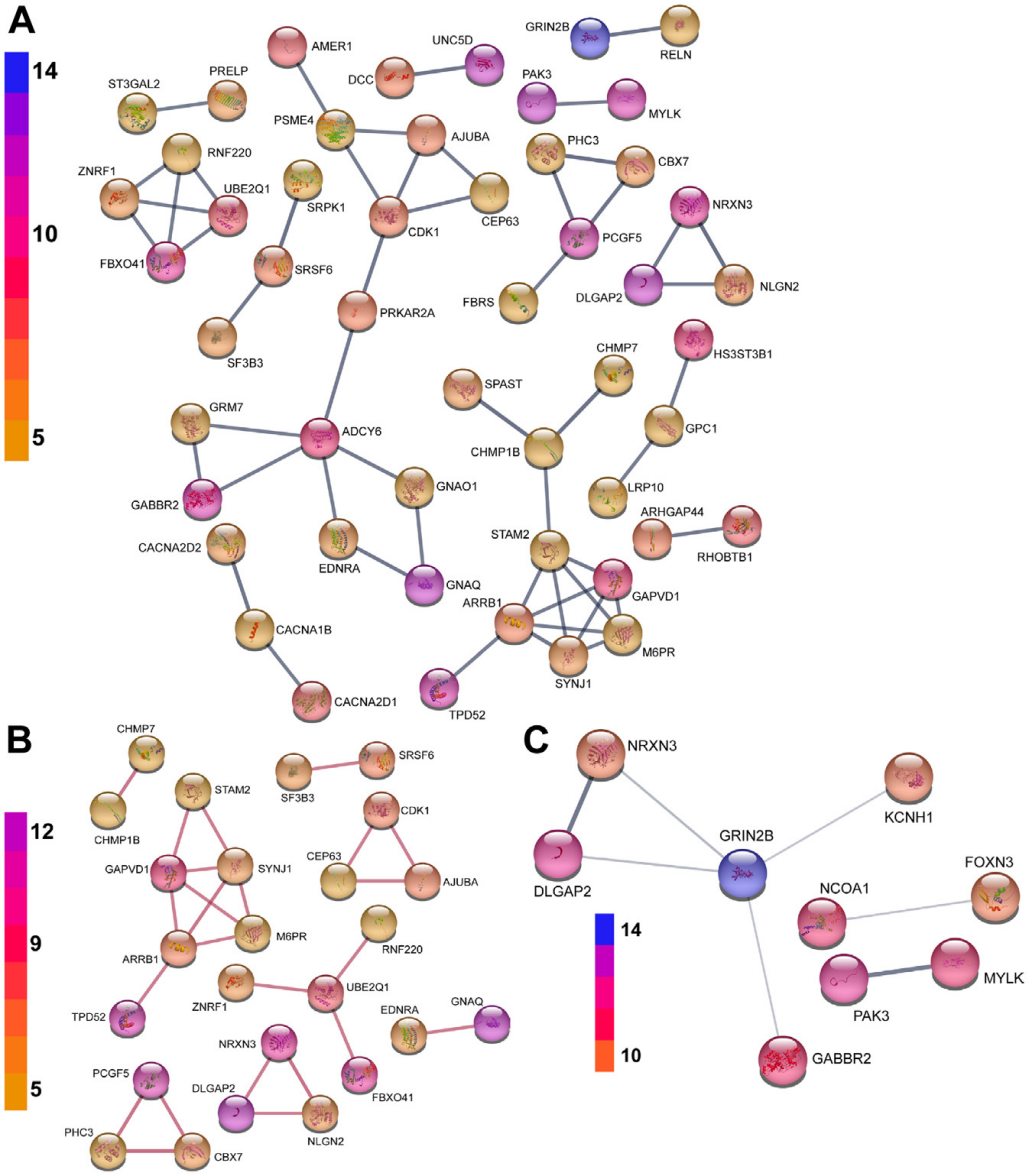
**Stringent analysis of TargetScan data identifies clusters of proteins and consequent pathways potentially manipulated by induction of miRNAs during HCMV infection of CD34+ cells**. *A* and *B*, Genes predicted to be targeted by at least five miRNAs by TargetScan were analyzed *via* STRING for protein–protein interactions. Functional (*A*) and physical (*B*) interactions were limited to only those of high (>0.9) confidence, and only proteins that interacted with at least one other protein at this level are shown. The number of targeting miRNAs is indicated by the relative coloration. (*C*) Protein–protein interactions (confidence >0.4, the thicker connecting line indicates higher confidence) for those genes predicted to be targeted by at least 10 miRNAs by TargetScan. HCMV, Human cytomegalovirus; miRNAs, microRNA.

While the majority of the identified proteins (135) displayed no associations at this level of confidence, the remaining 51 proteins formed 12 distinct groups, with between 2 and 12 proteins in each (Fig. 5*A*). One group of particular interest was formed of the genes ring finger protein 220 (RNF220), zinc and ring finger 1 (ZNRF1), F-box protein 41 (FBXO41) and ubiquitin conjugating enzyme E2 Q1 (UBE2Q1). These form an extremely high confidence cluster (pairwise confidence for association between each hit ≥0.9, on a scale 0–1), while only presenting low confidence (<0.4) interactions with other identified target genes. When altering the STRING-analysis to be based on physical interactions only, the resulting plot reflects curated data that indicate that RNF220, ZNRF1, and FBXO41 are all thought to physically interact with UBE2Q1 (Fig. 5*B*). As UBE2Q1 is an E2 ubiquitin ligase (41), and the others are either E3 ubiquitin ligases (RNF220, ZNRF1) (42, 43, 44) or a component of E3 ubiquitin ligase complexes (FBXO41) (45), this makes biological sense. Importantly, these four genes have all previously been shown to be expressed throughout the myeloid progenitors by RNA-seq (Table S5) (46). Furthermore, ZNRF1 has recently been shown to regulate ligand-induced epidermal growth factor receptor (EGFR) signaling, with loss of ZNRF1 resulting in increased susceptibility to HSV-1 infection (47). From an HCMV standpoint, entry is dependent on sustained EGFR signaling in both CD34+ cells and monocytes (48, 49) and thus the downregulation of ZNRF1 *via* miRNAs may contribute to this and enhance the establishment of latency.

### Upregulated miRNAs target multiple chromatin-modifying enzymes

The STRING analysis revealed another group of interesting target genes (polyhomeotic-like protein 3, chromobox protein homolog 7, polycomb group RING finger protein 5, and probable fibrosin-1) which are primarily associated with histone modification *via* a protein regulator of cytokinesis 1 (PRC1)-like complex (50, 51, 52, 53). Again, these four genes have previously been shown to be expressed throughout the myeloid lineage (although chromobox protein homolog 7 at a fairly low level). During both lytic and latent infection, the incoming HCMV genome is rapidly associated with histone proteins that are post-translationally modified to promote the silencing of the MIEP and lytic gene expression (54). Once again, the prediction would be that these silencing genes would be downregulated by the changes in miRNA expression and thus could be considered pro-viral. In lytic infection, this could be explained as a viral countermeasure to potential silencing, but in latency, this explanation is less valid (since viral lytic gene expression is silenced). However, this is predicated on the assumption again that the effects are direct—that is, targeted to the viral genome directly whereas changes in PRC1-like complex activity may regulate host gene expression much akin to the same concepts discussed in the context of LSD1. Indeed, the regulation of the MIEP during HCMV latency has been linked with the activity of heterochromatin protein 1 (55, 56) and PRC2 (57)—whereas in lytic infection it was demonstrated that PRC1 KO was detrimental to viral replication (58) and thus loss of PRC1 would be pro-latency under this criterion.

If we increased the stringency of our analysis further, limiting ourselves to proteins predicted to be targeted by at least 10 upregulated miRNAs, a list of 38 proteins was returned (Table S4). STRING analysis reveals a cluster of five genes (Fig. 5*C*), dominated by a number of genes with reported roles in neuronal biology, along with two pairs of genes: nuclear receptor coactivator 1 and forkhead box protein N3, and serine/threonine-protein kinase PAK with myosin light chain kinase, smooth muscle. Although the higher stringency yielded limited extra information, an interesting pair of genes emerged, bromodomain adjacent to zinc finger domain protein 2A and PH-interacting protein, which are both highly expressed throughout the myeloid lineage and contain bromodomains (59, 60). Recent work has demonstrated that bromodomain proteins regulate HCMV latency and could be an important target for the pharmaceutical elimination of HCMV (61). Thus, the manipulation of these proteins during the early stages of infection is consistent with the role of this family of proteins in HCMV biology.

### Targeting of a dense cluster of entry and trafficking-associated functions

The most densely connected cluster revealed by our more stringent analysis contains a core formed of signal transducing adapter molecule 2 (also connected to charged multivesicular body protein 7 and spastin *via* charged multivesicular body protein 1B), GTPase-activating protein and VPS9 domain-containing protein 1 (GAPVD1), beta-arrestin-1 (ARRB-1) (also connected to tumor protein D52), synaptojanin-1, and cation-dependent mannose-6-phosphate receptor. Expression of all these genes has been detected throughout the myeloid lineage. All the proteins encoded by these genes are connected to vesicle formation and trafficking, and many are associated with the cellular endosomal sorting complex required for transport machinery. Focusing on the core, a completely interconnected group of 5 (ARRB-1, synaptojanin-1, cation-dependent mannose-6-phosphate receptor, GAPVD1 and signal transducing adapter molecule 2), we investigated their potential connection to the establishment of HCMV latency.

Beta-arrestin-1, encoded by ARRB-1, regulates agonist-mediated G-protein coupled receptor (GPCR) function. In particular, this protein can bind to activated, phosphorylated GPCRs and thus prevent it from binding to its cognate G-protein (62). They can also act as clathrin-associated sorting proteins and thus drive the internalization and functional downregulation of a wide range of GPCRs. Crucially for HCMV infection, particularly in sites of latency, beta-arrestins can act as a signaling scaffold for mitogen-activated protein kinase pathways and drive extracellular signal-regulated kinase 1/2 activation, which has been shown to be a key step in ensuring the survival of virally infected CD34+ cells during the very early stages of infection (63). While downregulation of ARRB-1 is likely to have a range of effects upon the cell, this could be part of the effort to stymie the pro-HCMV signalosome activated by the virus and thus allow the infected cell to undergo apoptosis.

Another key signaling pathway strongly linked to HCMV infection is the epidermal growth factor pathway, which can lead to activation of and be regulated by extracellular signal-regulated kinase 1/2 activity. While the role of EGFR signaling during lytic infection of specific cell types has been contested, there is a growing body of evidence for its role in latency, particularly surrounding early entry events (48, 64). GAPex-5, the protein encoded for by GAPVD1, is an endosomal protein that can regulate the function of Rab5 *via* its guanine exchange factor activity (65), and Rab5 has been shown to regulate the early stages of EGFR-trafficking (66). Down-regulation of GAPex-5 levels by upregulation of GAPVD1-targeting miRNAs would be predicted to inhibit the degradation of activated EGFR and lead to its retention in early endosomes, from whence it could continue to signal, in all likelihood to the relative benefit of the virus based on our current understanding of viral entry into myeloid cells (64). This effect may be potentiated by the simultaneous targeting of STAM-2, which can also function to downregulate EGFR signaling (amongst other receptor tyrosine kinases), and thus ensure this key signaling pathway remains active for as long as possible, to ensure the correct trafficking of the virus while also impacting on cellular survival.

### A cluster of voltage-gated calcium channel subunits are targeted by upregulated miRNAs

Another cluster with intriguing biology in the context of HCMV was the identification of a cluster with a role in the regulation of calcium signaling. Indeed, during lytic infection, HCMV has been shown to trigger the depletion of intracellular Ca^2+^ stores in both fibroblasts and neuronal progenitors (67, 68). For potential roles during latent infection, it is also well established that calcium signaling is a key trigger of apoptosis. During entry into sites cells non-permissive for lytic infection (*e.g.* CD34+ cells and monocytes) and thus potential sites of latency/persistence, HCMV gene expression is highly restricted with limited viral anti-apoptotic genes expressed. As a result, HCMV triggers host-encoded survival pathways to counteract the simultaneous activation of cell death pathways *via* infection (63, 69). Consequently, it will be interesting to test the hypothesis that an important target could be calcium signaling and associated cell death. A cluster of three genes encoding calcium channel subunits, namely, voltage-dependent N-type calcium channel subunit 1VB, CACNA2D1, and CACNA2D2 were revealed by this analysis. Peptides from all three have been detected in CD34+ cells, while CACNA2D1 and CACNA2D2 have each been detected at the transcriptional level, albeit inconsistently between datasets (46, 70). Given that simple overexpression of CACNA2D2 is sufficient to trigger apoptosis in a range of cell types (71), these would make attractive targets to attempt to block the initiation of apoptosis as the virus navigates the host cell environment to establish latency.

**Figure 6:**
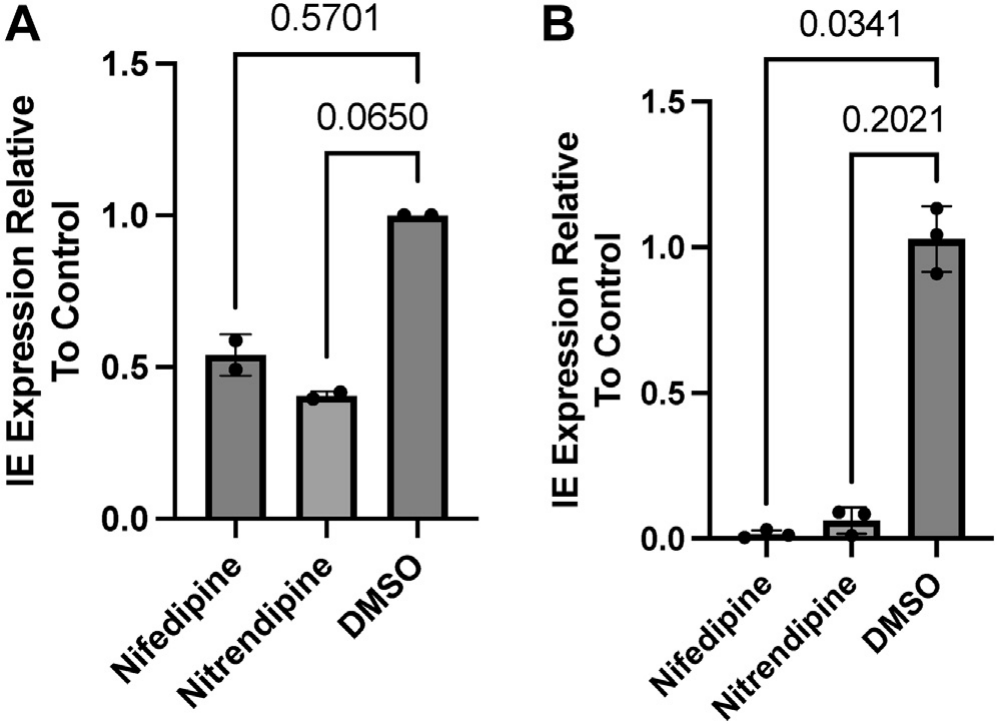
**Calcium channel inhibitors prevent expression from the MIEP selectively during reactivation.***A*, THP-1 cells were infected at MOI = 5 with HCMV strain Merlin. At 1hpi, cells were treated with calcium channel inhibitors or solvent control. At 24hpi, RNA was harvested, and total IE gene expression was quantified by qRT-PCR (n = 2 independent replicates). *B*, Primary CD14+ monocytes were infected at MOI = 5 with HCMV strain Merlin. After 3 days, differentiation into MoDCs was initiated with IL-4 and GM-CSF. Six days later, cells were treated with calcium channel inhibitors for 3 h prior to IL-6 treatment, and viral IE gene expression was quantified by qRT-PCR 24h later (n = 3 independent replicates). Summary data shown as mean±SD. Statistical comparison by one-way ANOVA with non-parametric Kruskal–Wallis test with Dunn’s multiple comparison correction. GM-CSF, granulocyte/macrophage-colony stimulating factor; HCMV, Human cytomegalovirus.

One anticipated goal of the *in silico* approach taken was to take a different route to the identification of pathways potentially important in latency and reactivation. At the molecular level, the differential regulation of MIE gene expression is a determinant of latency (repression) and reactivation (activation)—for example, differential regulation of chromatin underpins the latency/reactivation switch. The identification of chromatin-modifying enzymes being a target for miRNAs led us to consider whether other functions being targeted during these early stages may have a role in the regulation of viral gene expression. Thus we tested whether calcium signaling could have a role in HCMV latency *in vitro*. To do this, THP1 cells were infected with HCMV and then incubated with calcium channel inhibitors 1hpi and then analyzed for IE gene expression after 24h. At 24hpi, a small but reproducible effect on transient IE gene expression was observed in the presence of the inhibitors (Fig. 6*A*). One interpretation of these data was that calcium channel activity may enhance MIE gene expression and thus we hypothesized that this may also be true in reactivation where MIE gene expression must be induced – with analogy to the use of histone modifying agents. Thus, MoDCs were generated from infected CD14+ cells and then incubated with the same Ca2+ inhibitors, and then stimulated with IL-6 to promote reactivation. The data show that inhibition of Ca2+ activity dramatically reduced IL-6–induced reactivation (Fig. 6*B*).

## Final discussion

Here we report a dataset that captures changes in the miRNAome of virally infected CD34+ cells during the initial stages of viral infection. The dataset is presented as a resource for investigators which may provide a springboard for future investigations or provide the basis to partially explain observations colleagues in the field may have observed. In presenting the dataset we demonstrate some of the *in silico* approaches we have taken for analysis of the dataset and give examples (through both experimental and published data) of how this dataset can be interrogated to trigger new lines of inquiry.

It is of course important to highlight that the *in silico* analyses are based on predicted changes in protein expression induced by miRNAs and thus would require independent validation. The drive behind the approach undertaken was to try and shift away from the “one gene one miRNA” approach but instead to use the changes in the miRNAome as an indicator of potential pathways and hubs that could be of importance in HCMV biology—but hubs that perhaps require subtle modification of activity rather than gross changes in function—changes that we predict miRNAs would be capable of inducing. In essence, the predictions are based on the principle of the “Goldilocks phenomenon” that pervades biology (72, 73, 74, 75, 76, 77). For instance, our observations studying knockout *versus* transient knockdown of JDP2 during different stages of viral infection in the myeloid cells reflect on how the context of the activity is important but may also reflect differences between a reduction *versus* a complete loss of activity in the cells. Indeed a prior study of EBV and JDP2 revealed that knockdown and overexpression of JDP2 did not have directly opposite effects on EBV latency and reactivation.

Another example of context-dependent roles was revealed by the calcium channel data. It is highly plausible that mechanisms that contribute to the silencing of the MIEP during latency are required to be disabled for HCMV reactivation (where the MIEP is activated). This concept is evident in the many studies that have documented the impact of epigenetic modifiers on herpes virus latency and reactivation (61, 78, 79). Thus, the data argue that the MIEP is sensitive to calcium ion channel activity. Transient downregulation of Ca^2+^ ion channel activity may contribute to MIEP silencing in latency but also argues that the activation of this pathway is important for reactivation. We have previously demonstrated that HCMV reactivation in MoDCs is cyclic AMP-responsive element-binding protein transcription factor–dependent (80). It has been shown that Ca2+ activity can potentiate cyclic AMP-responsive element-binding protein activity in a mitogen-activated protein kinase-dependent manner (81, 82) thus, it is possible that the role of Ca2+ ion channels in reactivation is to augment this pathway important for reactivation in dendritic cells (32, 83). More generally, it demonstrates that events that happen during the establishment of latency have the potential to illuminate functions that could be important for reactivation. Put simply, a number of events that occur during the establishment of latency require reversal for reactivation.

Another important aspect of the work was taking the in-silico data and anchoring it in the context of both HCMV and cell biology. For instance, “neuron differentiation” was the most highly enriched pathway in our earlier analysis and thus we investigated what genes associated with neuronal functions may be identified in our more stringent analysis and thus impart this neuronal signature. This revealed GRIN2B (encoding GluN2B), the most heavily targeted gene identified, formed a strong association with RELN (encoding Reelin). GluN2B is a component of *N*-methyl-D-aspartate receptor complexes, which act as ligand-gated ion channels for the propagation of neuronal signals *via* synapses, while Reelin is an extracellular serine protease associated with a wide range of neurological functions, including regulation and clustering of *N*-methyl-D-aspartate receptors (84, 85, 86). While one could speculate as to how these proteins may act in a non-neuronal cell and be connected to infection by HCMV, possibly by impacting the entry process of the virus, there exists very limited evidence for their expression in myeloid progenitor cells. Neither GRIN2B nor RELN was found to be expressed in multiple RNA-seq datasets (46, 70), and only a single Reelin peptide was only detected by mass spectrometry in less than 10% of samples studied, while GluN2B went undetected (70). There remains the possibility that these proteins could be produced in response to infection, but they do not seem likely biologically relevant targets for the early stages of HCMV infection of CD34+ cells, and thus it remains critical to cross-reference these predictions with the wealth of transcriptional datasets also available. Indeed, this was another driver in our decision to publish our own dataset and make it available.

Another important point from the identification of the neuronal differentiation signature is that also revealed a second cluster of neurologically related genes. NRXN3, DLGAP2, and NLGN2 also formed a cluster with associated functions and also with evidence of physical interactions. They encode for neuroligin-2, Disks large-associated protein two and neurexin-3, respectively, and are thought to be associated with cell–cell interactions, intercellular signaling, and synapse function (87). Importantly, there is at least some evidence of transcription of all 3 cluster members in myeloid progenitor cells and thus although their identification and understanding are derived from the disease state/cell type they were first characterized in, it does not rule out it may have important functions in other cells and should not be dismissed out of hand. Indeed, there are many examples of genes identified through the disease states they are involved in rather than their normal physiological role. Our own studies of the viral beta 2.7 RNA and interaction with GRIM-19 is a prescient example of this where the RNA targets GRIM-19 to sustain its normal physiological role rather than prevent a specific retinoic acid phenotype it is responsible for in cancer cells (and how it is was named) (88, 89, 90, 91).

In summary, we present a dataset coupled with examples of *in silico* analyses that can be performed to interrogate this—approaches that we decided to utilize in response to the unprecedented pandemic situation in 2020. The dataset reveals changes in the miRNAome that occur upon infection of primary CD34+ hematopoietic cells with HCMV. They reflect a very discrete set of changes and likely reflect both pro and anti-viral responses that are important for, and the restriction of, viral infection and establishment of latency and, by extension, allowed us to identify a potential role for Ca2+ ion channel signaling in HCMV reactivation.

## Experimental procedures

### Cell culture and virus

CD34+ cells (Lonza) and CD14+ monocytes were maintained in X-VIVO 15 serum-free media (Lonza), human fetal foreskin fibroblasts (HFFs) and 293T cells were maintained in Dulbecco's modified Eagle's medium (DMEM) (Gibco), THP-1 cells were maintained in RPMI 1640, and ARPE-19 cells were maintained in DMEM/F-12 (Gibco). DMEM, DMEM/F-12, and RPMI 1640 were all supplemented with 10% FBS. CD14+ cells were obtained from healthy volunteers (NHS London Hampstead research ethic committee - 08/H0720/46) by initial centrifugation of PBS-diluted whole blood over Histopaque-1077 to isolate PBMCs, followed by positive magnetic selection (CD14+ microbeads, MS columns, Miltenyi Biotec). All cells were maintained at 37 °C with 5% CO_2._ HCMV strains Merlin and TB40/E were passaged through ARPE-19 cells to maintain broad cellular tropism before final amplification in HFFs, while VR1814 was amplified directly in HFFs. HCMV was UV-inactivated using a 2UV Transilluminator (UVP), with sufficient UV exposure for inactivation determined as the length of exposure required to reduce the number of IE-positive HFFs to <1% observed with untreated HCMV 24hpi.

### MicroRNA screen

CD34+ cells were mock infected or infected with a myelo-tropic stock of the Merlin isolate of HCMV (MOI = 5, results in 15% IE-positive dendritic cells 24hpi). Three hours later, the inoculum was removed and replaced with fresh media, and a further 3 h later cells were lysed and RNA isolated using a miRNA isolation kit (Qiagen). RNA was then sent to Qiagen for analysis of expression of 1055 cellular miRNAs (by Qiagen Human miScript miRNA Array V16.0) and expression in mock and infected CD34+ cells was used to identify changes in miRNA expression (technical n = 1, biological n = 3). *P* values were calculated by Student’s *t* test.

### MicroRNA extraction and cDNA synthesis for targeted miRNA validation

CD34+ cells, CD14+ monocytes, or HFFs were infected at MOI = 5 with HCMV strain TB40/E or Merlin. After 6 h, small RNAs (<200 nt) were extracted by PureLink miRNA Isolation Kit (Two column method, Thermo Fisher). Samples were subjected to first-stand cDNA synthesis as per Cirera and Busk, 2014 (92). Briefly, equal quantities of small RNA per sample (15–50 ng) were mixed with *E. coli* poly(A) polymerase reaction buffer (1×), 100 μM ATP, 100 μM dATP, 100 μM dCTP, 100 μM dGTP, 100 μM dTTP, 1 μM universal RT primer (CAG GTC CAG TTT TTT TTT TTT TTT VN), 100U M-MuLV reverse transcriptase and 1U *E. coli* poly (A) polymerase. Samples were incubated at 42 °C for 1 h followed by reaction inactivation by 5 min at 90 °C. Samples were diluted 4-fold with RNase-free water.

### Total RNA extraction and cDNA synthesis

Total RNA was extracted by Qiagen RNeasy kit, using columns from Epoch Life Sciences, according to the manufacturer’s protocol. cDNA was synthesized from an equal quantity of RNA per sample using the Qiagen Quantitect Reverse Transcription kit as per the manufacturer’s instructions.

### Quantitative PCR

miR-specific primers were designed using the miRprimer software (Table S6) (93). Each qPCR reaction contained 1× PowerUp SYBR Green master mix, 500 nM forward and reverse primer and an equal quantity of diluted cDNA. Cycling conditions were 2 min at 50 °C, 2 min at 95 °C followed by 50 cycles of 95 °C for 10 s and 60 °C for 60 s.

Relative quantification of 18S, UL138 and total IE transcripts was performed with 1× PowerUp SYBR Green master mix with 250 nM of forward and reverse primers, using cycling conditions of 50 °C for 2 min, 95 °C for 2 min and 40 cycles of 95 °C for 15 s, 60 °C for 15 s and 72 °C for 1 min. The gene-specific primers employed were: UL138 (F, GAG CTG TAC GGG GAG TAC GA; R, AGC TGC ACT GGG AAG ACA CT), 18S (F, GTA ACC CGT TGA ACC CCA; R, CCA TCC AAT CGG TAG TAG CG), JDP2 (F, GGA GGT GAA ACT GGG CAA GA; GCT GCT GCG ACT TTG TTC TT), gB (F, GAG GAC AAC GAA ATC CTG TTG GGC A; R, TCG ACG GTG GAG ATA CTG CTG AGG) and total IE (F, GGA CCC TGA TAA TCC TGA CG; R, ATC TTT CTC GGG GTT CTC GT).

All qPCR reactions were performed and analyzed using the QuantStudio 3 (c) system. All qPCR reactions were performed in technical duplicate, with biological replicates as noted in the appropriate figure legend.

### Infection assays in sites of latency

100,000 JDP2 or control knockout THP-1 cells were infected at MOI = 5 with HCMV strain TB40/E in medium supplemented with 2% serum. One day post-infection, cells were washed with PBS and RNA extracted as described, or media was replenished, prior to RNA extraction 3 days post-infection or further maintenance for assessment of HCMV reactivation. RNA was processed and analyzed by qRT-PCR as above, with quantification by the ΔΔCt method. HCMV reactivation was assessed by treatment of infected THP-1 cells with 50 nM phorbol 12-myristate 13-acetate (PMA, Cayman Chemical) 5 days post-infection. HFFs were overlaid 24h later, and cells fixed in ice-cold ethanol and stained for viral IE (6F8.2; Merck Millipore; 1:2000 dilution and goat anti-mouse IgG–Alexa-fluor 568 (Life Technologies; 1:2000 dilution)) protein expression. IE+ foci were visualized on a Leica DMI4000B widefield fluorescent microscope and counted manually. Statistical comparisons were all performed *via* the non-parametric Kruskal-Wallis test in GraphPad Prism version 9.5.1.

In all, 250,000 CD14+ monocytes were infected at MOI = 5 with HCMV strain VR1814. One day post-infection, the cells were washed with PBS and media replenished, with 1000U/ml interleukin-4 (IL-4, Peprotech) and granulocyte/macrophage-colony stimulating factor (Peprotech) added after a further 2 days to induce differentiation into dendritic cells. Cells were maintained for an additional 4 days prior to transfection with 50pmol control (Control siRNA-A, sc-37007, Santa Cruz Biotechnology) or JDP2 (sc-38017, Santa Cruz Biotechnology) siRNA with Lipofectamine 2000 (ThermoFisher) as per manufacturer’s protocol. 48h post-transfection, cells were harvested for RNA or treated with 50 ng/ml recombinant interleukin-6 (IL-6, Peprotech). RNA samples harvested from IL-6-treated cells 24h post-stimulus. RNA was processed and analyzed by qRT-PCR as above.

### Impact of EIPA treatment on stimulation of miRNAs

250,000 THP-1 cells were treated with 15 μM 5-(N-Ethyl-N-isopropyl)amiloride (EIPA) for 3 h prior to infection at MOI = 5 with HCMV strain Merlin. RNA or DNA was harvested at 6hpi. miRNA was isolated and analyzed by qRT-PCR as above. DNA was harvested by Nonidet-P40 lysis as previously described (94) and analyzed by qPCR using primers directed against viral gB. Statistical comparisons were performed *via* the non-parametric Mann–Whitney U test in GraphPad Prism version 9.5.1.

### Assessment of calcium channel inhibitors on IE gene expression during infection and reactivation

In all, 250,000 THP-1 cells were infected with HCMV strain Merlin at MOI = 5, then treated with 10 μM Nifedipine (Sigma-Aldrich), 5 μM Nitrendipine (Sigma-Aldrich) or DMSO 1hpi. RNA harvested 24hpi and analyzed by qRT-PCR for IE gene expression as above.

250,000 CD14+ monocytes were infected at MOI = 5 with HCMV strain Merlin and differentiated into monocyte-derived DCs as above. Cells were treated with 10 μM Nifedipine, 5 μM Nitrendipine (kind gifts from Jamal Mankouri, University of Leeds) or DMSO for 3h prior to stimulation with 50 ng/ml IL-6 as above. RNA was harvested 24hpi and analyzed for IE gene expression as above. Statistical comparisons were all performed *via* the non-parametric Kruskal–Wallis test with Dunn’s multiple testing correction in GraphPad Prism version 9.5.1.

### *In silico* analysis of miRNA data

To perform pathway enrichment analysis directly upon the miRNAs, upregulated miRNAs were submitted to the TAM2.0 (http://www.lirmed.com/tam2/) webserver (23, 24), with the list of all quantifiable miRNAs being used as background. Prediction of protein targets was performed using the MIENTURNET (http://userver.bio.uniroma1.it/apps/mienturnet/) webserver (25). *p*-values calculated by both the TAM2.0 and MIENTURNET webserver employ the hypergeometric test (95). Protein-protein network interactions were analyzed *via* upload to the STRING database (https://string-db.org/), version 11.0 (40). Protein–protein interaction images were exported from STRING and modified for clarity and labeling in Adobe Illustrator.

### Generation of *JDP2* knockout cell line

A clonal THP-1 cell line stably expressing Cas9 was transduced with lentiGuide-Puro (A gift from Feng Zhang, Addgene plasmid #52963 (96)) vectors encoding sgRNA sequences targeting JDP2, or β_2_m or a scrambled sequence as controls, to generate three independent JDP2 knockout cell lines. Sequences of the sgRNAs were designed using the CRISPick tool from the Broad Institute (JDP2ko-1: TTG GTA TAC AGG AAT CCG AG, JDP2ko-2: TGT GCC CTC ACA GCT AGA TG, JDP2ko-3: AGG GTG CAA TCA TGG CCC CG, Scramble: GCA CTA CCA GAG CTA ACT CA).

Oligonucleotides encoding sgRNAs targeting JDP2, β_2_m, or scrambled were designed as described by the original authors (96, 97). Pairs of oligonucleotides were annealed and phosphorylated using T4 polynucleotide kinase (New England Biolabs) and ligated into gel-purified (Qiagen QIAquick gel extraction kit), dephosphorylated (Antarctic phosphatase, New England Biolabs), *BsmB*I-linearized lentiGuide-Puro with T4 DNA ligase (New England Biolabs) as per manufacturer’s instructions. Ligated plasmids were transformed into One Shot TOP10 Chemically Competent *E. coli* (Invitrogen) as per the manufacturer’s instructions and plated onto LB agar supplemented with 100 μg/ml ampicillin. Single colonies were inoculated into 100 μg/ml ampicillin-supplemented LB, grown to stationary phase, and plasmid purified by QIAprep Spin Miniprep kit (Qiagen). Insertion of sgRNA sequence was confirmed by Sanger sequencing (Eurofins).

Lentiviral vectors were produced from 293T cells transfected with a 3:2:1 (by mass) ratio of sgRNA-bearing lentiGuide-puro vector, pCMV-dR8.91 and pMD2.G with 3 μl of TransIT-293 (Mirus) transfection reagent. Supernatants were collected 2 days later, clarified by centrifugation, and spinoculated onto THP-1 cells. Selection was induced by puromycin treatment 2 days post-transduction and maintained for 7 days. Cells were expanded for use in described assays.

### Functional assessment of miRNA mimics on JDP2 expression

Due to the size of the JDP2 3′ UTR (4.6kb), it was commercially cloned and split over two luciferase-expressing vectors referred to as pJDP2-A and pJDP2-B (GeneCopoeia, HmiT130565, Supplementary Data 1). Sub-confluent HEK293T cells in a 96-well plate were transfected with control (pControl, GeneCopoeia, CmiT000001-MT05) or JDP2-A or JDP2-B plasmids (10 ng/well) plus 20pmol miRNA mimics (mirVana, ThermoFisher) of either hsa-miR-9-5, 137-5p or 218-5p, or a combination of all three mimics (60pmol), or a negative control mimic (mirVana Negative Control #1) using 0.5 μl Lipofectamine 2000 (ThermoFisher) per well, with transfection conditions as per manufacturer’s instructions. Twenty 4 hours later, media was removed from the cells and the cells lysed with Gaussia luciferase reagent and after 2 min the luciferase signal was calculated. Statistical analysis by two-way ANOVA with Tukey’s multiple comparison test in GraphPad Prism version 9.4.1.

To test for impact on JDP2 RNA expression, HEK293T or THP-1 cells were transfected with miRNA mimics of either hsa-miR-9-5, 137-5p or 218-5p, or a combination of all three mimics (60pmol), or a negative control mimic as previously. After 24 h, RNA was isolated and analyzed by qRT-PCR for JDP2 expression as above. Statistical analysis was done by Kruskal–Wallis test with Dunn’s multiple testing correction in GraphPad Prism version 9.5.1.

## Data availability

The data supporting the findings of this study are available in the methods, results and supplementary material associated with this article.

## Supporting information

This article contains supporting information (Figures S1–S4, Tables S1–S6, and Supplementary Data 1) (46).

## Conflicts of interest

The authors declare that they have no conflicts of interest with the contents of this article.
