# Supplementary material for: In silico interrogation of the miRNAome of infected hematopoietic cells to predict processes important for human cytomegalovirus latent infection
Source: J Biol Chem. 2023 Apr 19;299(6):104727. doi: 10.1016/j.jbc.2023.104727 (PMC10206818; doi:10.1016/j.jbc.2023.104727)
Supplement: Supporting Information [file mmc7.pdf]

Supporting Information for:

In silico interrogation of the miRNAome of infected haematopoietic cells to predict processes important for human cytomegalovirus latent infection

*Murray, M. J., Bradley, E., Ng, Y., Thomas, O., Patel, K., Angus, C., Atkinson, C. & *Reeves, M. B.

Supporting Information contents:

Within this file:

1. Supplementary Figure 1
2. Supplementary Figure 2
3. Supplementary Figure 3
4. Supplementary Figure 4
5. Supplementary Data 1

Additional supporting data, separate to this file:

1. Supplementary Table 1
2. Supplementary Table 2
3. Supplementary Table 3
4. Supplementary Table 4
5. Supplementary Table 5
6. Supplementary Table 6

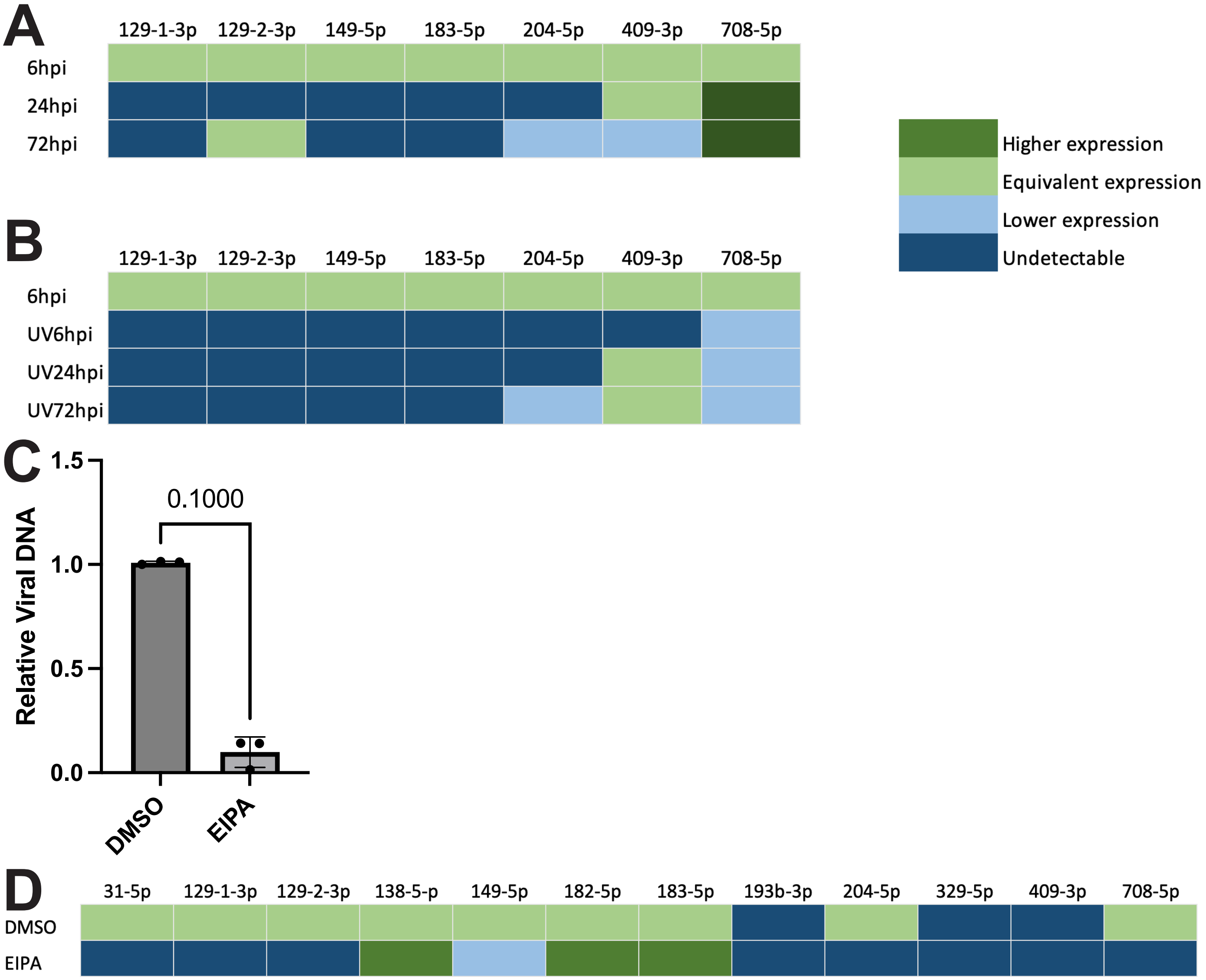

Supplementary Figure 1: miRNA induction varies over time, in response to UV-inactivated virus and in response to inhibition of infection with EIPA.
(A,B) CD34+ cells were infected at MOI=5 with HCMV strain Merlin or UV-inactivated HCMV. At 6, 24 and 72hpi miRNA expression was quantified by qRT-PCR (n=1). Data is shown for detected miRNAs as expression compared to detected expression at 6hpi in HCMV infected cells in (A) HCMV-infected cells over time and in (B) cells infected with UV-inactivated HCMV over time. (C,D) THP-1 cells were treated with 15μM EIPA for 3 hours prior to MOI=5 infection with HCMV strain Merlin. (C) At 6hpi internalised DNA quantity detected by qPCR and expressed relative to DNA detected in DMSO-treated cells (n=3 independent replicates). Statistical comparison by non-parametric Mann-Whitney test. (D) miRNA expression at 6hpi was quantified by qRT-PCR (n=1). Data is shown relative to expression in DMSO-treated, infected cells. Summary data displayed as mean±SD.

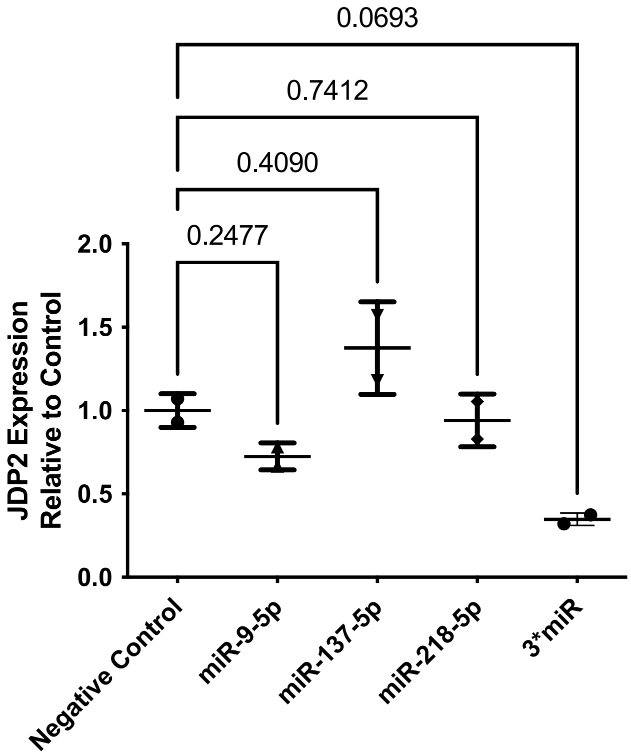

Supplementary Figure 2: JDP2 expression in THP-1 cells is reduced by transfection with multiple miRNAs

THP-1 cells were transfected with individual miRNA mimics or a combination of all 3 (3*miR). 24 hours later, JDP2 expression was enumerated by RT-qPCR and expressed relative to expression in negative control miRNA-transfected cells (n=2 indepedent replicates). Statistical comparison by 1-way ANOVA with non-parametric Kruskal-Wallis test with Dunn's multiple comparison correction, summary data displayed as mean±SD.

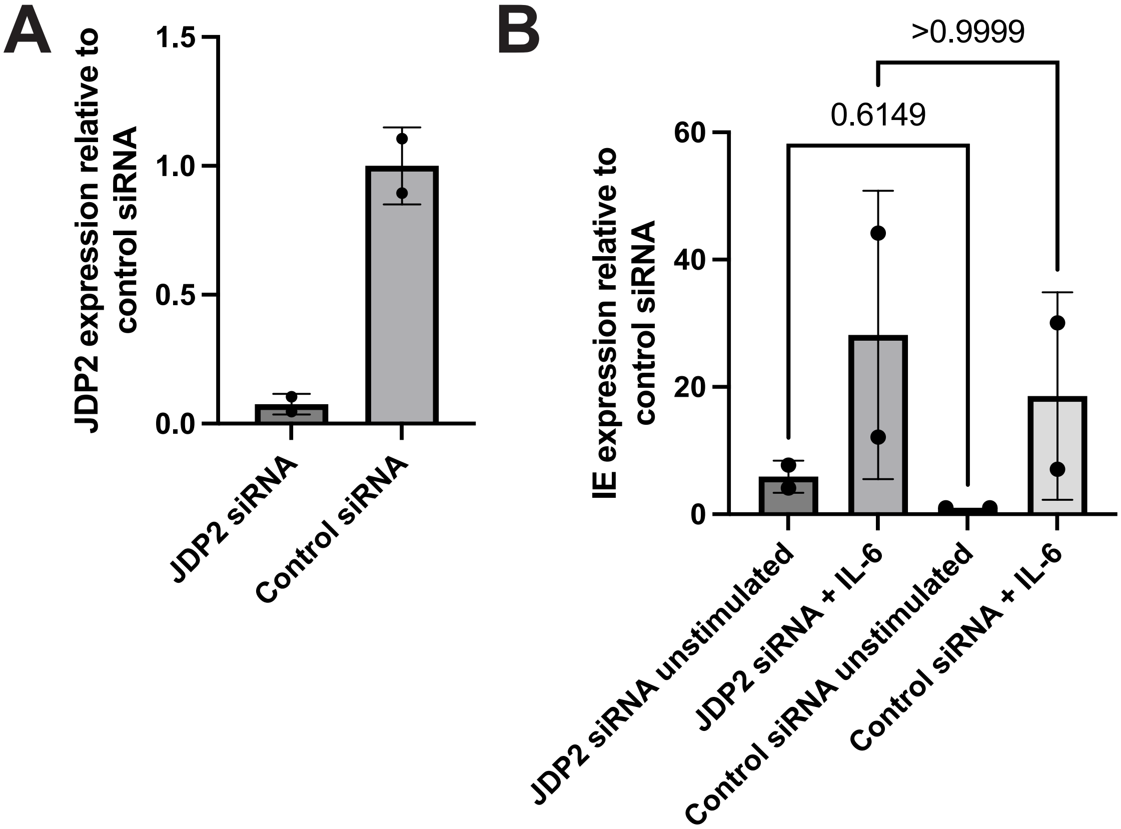

Supplementary Figure 3: Transient JDP2 knockdown increases IE gene expression in reactivating dendritic cells.

CD14+ monocytes were infected with HCMV and differentiated into dendritic cells with IL-4 and GM-CSF after 3 days. Cells were treated with control or JDP2 siRNAs 4 days later, and cells either harvested for RNA expression 2 days later, left untreated or stimulated with IL-6, prior to RNA harvest 24h post stimulation. (A) JDP2 expression at time of IL-6 stimulation was enumerated by qRT-PCR and expressed relative to control siRNA treated cells (n=2 independent replicates). (B) Total IE gene expression was assessed 24h post IL-6 treatment by qRT-PCR and expressed relative to control siRNA, unstimulated cells (n=2 independent replicates). Statistical comparison by 1-way ANOVA with non-parametric Kruskal-Wallis test with Dunn's multiple comparison correction. Summary data displayed as mean±SD.

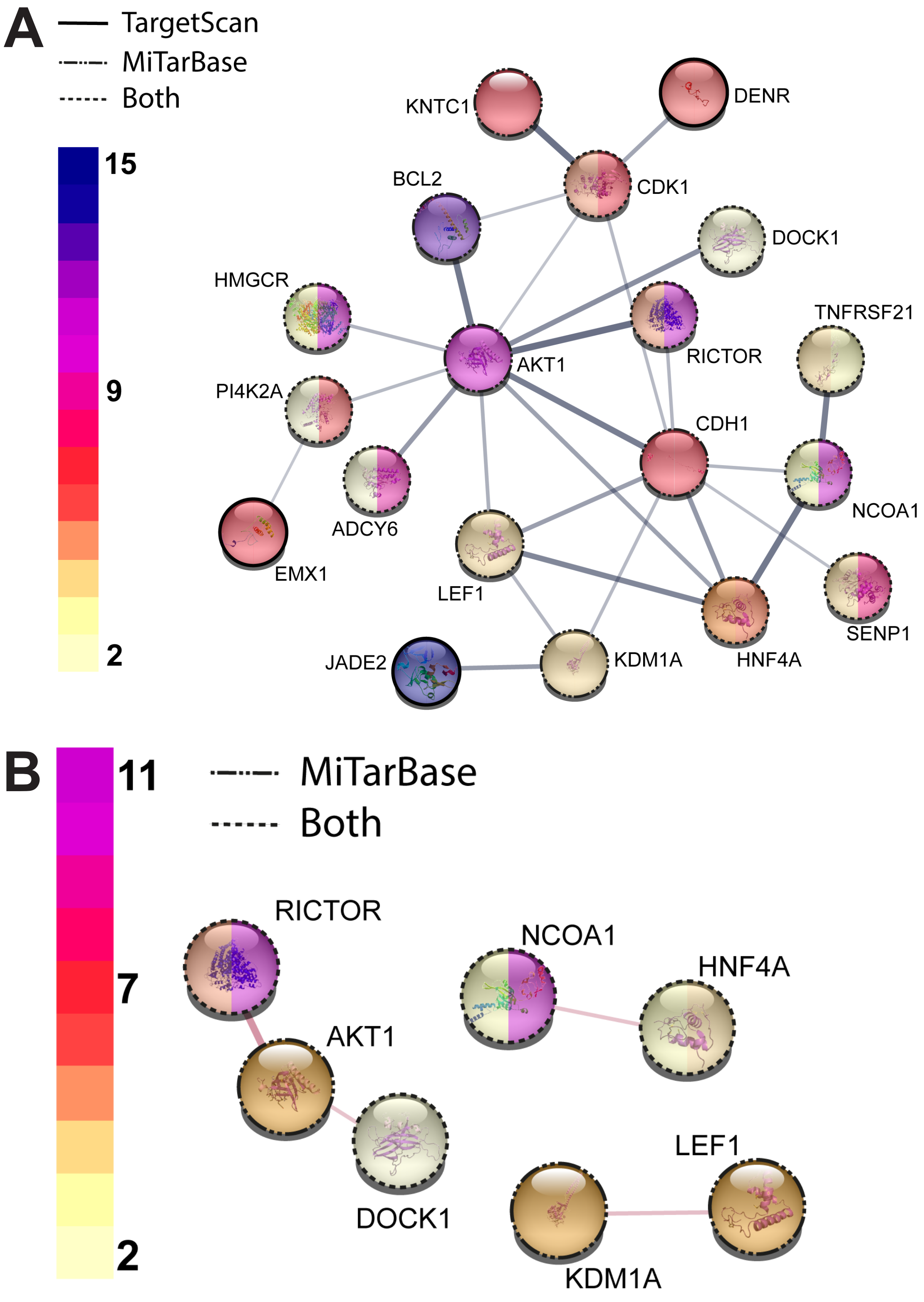

Supplementary Figure 4: Alternative display of protein-protein interactions generated by STRING

The initial list of 46 proteins was uploaded to STRING to assess functional (A) and physical (B) interactions, as per Fig 4. In this alternative display, identified genes are coloured according to the number of miRNAs predicted to target them. For genes identified in both databases, the left-hand colour represents the number of targeting miRNAs according to MiTarBase, whilst the right-hand colour represents the number according to TargetScan. The outline of each gene indicates which database(s) these genes were identified from.

Supplementary Data 1

pJDP2-A 3' UTR (-6~2388)
gaagtgaCCATGGGCTGGGAGGAGGTGGAGGAGGAGGAAGAGGAGAAGGAAAAGTGACGAAGAGAGAGGAGGAGGGG
GGCCCCAGATGGCCCTTCCTTTGGTGCATGAAAAACTGTACAATGAGGTTCAGCACAGCCAGCATCAGCCGAGCTTTTTG
TGAAACTCAGATCAGCCACCCAGGAGGAAGAGCGGGCTGAGGAAACCCAGAGGGACCAAGCGCTGAGACCAAAGTTGA
CCCTCGGGTAGGGTTGTCCTGCCTGGGGCCCCACTTGAAGGAGGCAGGACAGAGGCACCGAGGCCAGGGAGACGCCCA
ACGAGGCAGCCCTGGGCTCTTCTCTGGCCTCTTCACCAGGGCACCCATCCAAGGAACCTCCGAACAGCCAGGAAAAGCCA
TGAGTTGCAACCAAAACGCGGCTGAGGATGGAACTCAGAATGAAACTGCAACCCACCTGCCCCCAGCCCTGCCCCTCGCC
CTGATGCGAAGCTGGAGAGGGGCGTGCTGCGGGGCCCTGATGCCCCCACCCACCTCGGTCCAGCGCGGCCCTGCCCAGG
AGGCGGCAGCCGGGCGCACCCTCGCCAGCCCTGCTGGAGTTTGCTGTGGGCACTGAGGCGCGGGCGCCCTTCCAAAGCA
CATACTCACCGAATGTTTACAGACTGGCTGTCCTGGCAGGGCTTTCAACTGCACATGTTTTTTATACTTTCCTTTTTTTTTTTT
TTTTTAATATTTTTTACAAAAAAAAAGATTTTATACAAGCAATATATATATGGATTTCTATAATCACTCGATGTGATACAGTA
TAAATATGCTATGGTTTGTTTGTTATGAACAGATAGCCACCAGTTACGGCCGTTGTGTGTAACTCCTAAGTACTGTAGTCTC
TGGGTGTCGGGGGTGGCCAGGGCGGGGGCGGGGTGCATTTCCATCCTTGTAAACCCTTCATAGTACTCAGTCCTGTATCG
CTCAGTAAACATTGCTCTTACTTACATAGCCGCCTTGCGTGGTGTCTGCCTGGGGAATGGGTGCAAGGCCCTCTCAGGGTC
GGAGACTGTTTGGAGCCTCTGCTATAGGCCTGTTCATTTTCACAACAGCTCTCCTATTTCACAGATGAGGAAGCTAAGGCT
CAGAGACATTAAGCCACCTGCAGTTACTATTTTGAGAGCAGTCAGAGTGTTCGCTCACTCTGCAGATGTTTTCTGAGGGCC
TGTTGTGTGTGCCAGGCCCTGAGAATTCACTGGAGGATGAAACAGACCTAAGTCTTGCCCTTGTGGGGCCTCCAGTAAGG
AGTGAATCCTGTGCTTGCTCCATAAGTTGTCTCATTTCGTCTTCATGGCAGCCTTGCCAGGGAACAGCACTTGTCCCCAATT
CACAGATGGCGAATTAGAGGTCCAGGGAATTAAACTCGCAGACACCATTTGTTGAAGACTTATTTTGTGACAAGCAGACA
TAGTCTCTTCCCTTCTCACAAGAGCACAAACTAGGCACGATTCTTCTCATTTTACAGATGGTTTAACTGAGGCTCAGAGAA
GGTAAGCGACTTATCCAAGGTAACCCAGCTAGCAAGGGATAGAGCTGGTGTTTGAACTCTCCCTCCAGAGGCCTGTGACT
CTCCTCCTGTCACCAACCCACTATGATTGTCCTCATGGTTGAATAGGAAGCTTATGAGGCCACAGTTATAGATTTAGTTTCT
GGGAAGGCTGGATAGAGTTCCAGGTTGTCCCATGATCACAGAGTGAACCAACTACCCTGGTCAATGTGTGTCTGACCCAG
GGAAGGGTGTGGATGGCTTCTCTGCCCCCTCACCTCGACAGCTGAATCCCTGCCACCCAGGGCCGGAGCAGCTCAGTGGC
ATTCACTCAGTGCCTGCTGTGTACCTGGCACTCGGCAGTTCTCAAGGCATGTCACGCATGAGGAAGGCGGGAGAGGTGTT
TTTAGCTTCCTTGTATACTGAAGCTTGCAAAGGCTACAGTTTACCCAACTAAACTTGACTCCTGCATTCTAATCATGTGTTCT
TTCTCCTCCATCCCTGGACTTAGGATAACTAGTTGCTGGTGATGACTTCGTGTCAAGAACAGCAGTGAACCTGGGTTGCTT
CCCAAATTGTGGACGAGGCCTTCTGGGATGGGCAGAAGTGGAGAATGGTGATTAACACACCTGTGATCCCTCCCTCGCTC
ACTCCTCTCAGTTTCCCTCCACCCAACACACTTGGTGTGGACTCCCCTGCACCCCGCCCTAGAAACAGGACCTGGTCCTCTC
ACTGTCAGCTGTGGCAGAAGGTTGTGGCAGCGGGTTGCCTTCTGTCTCGGGGGTCTTGAGGCTGTCGGTCCGGACGATG
CAGGTGAGGCAGTGTCAGTTCTGCATCAGGAACCCCTCGCGCTGTAATCCTTTGATATTTT

pJDP2-B 3' UTR (2333~4643)
GAGGCAGTGTCAGTTCTGCATCAGGAACCCCTCGCGCTGTAATCCTTTGATATTTTTCAAGCCATGAGAAAAATGAAGTG
GACTTACCAGGGTCCCTCCAGTGACCTAGTGGATGTTCTTACAGGACTGCCGTGTGCCACTGGCTGTGGCTTCCCTGGTCC
TGTGCTTGGCCACCGGGAGCTGGCATCTCCAAAATAGAGCCTCCTTAGCCTGTGCAGGTGGGCTGTCTTCCAAAGTCACC
ACACCAGTGACTTGTGCTGTGCGGAGCCTTCCTCACCAGGACCACTGGCTGCTCCTGTGGCTAGAAGTCACAATTATAGG
CTTCATTGCAAGAGCCAGCATTTGAGTTTCTTCTGGGCACCTGCCACTGTGCATGTGTGACCTCAACTGCTTTGTGCACCCT
GACTGGGGGGTAGGTGCTAGCGTTCCAACATTACAGATGAGAAAACCGAGGCACACAGAAGAGGTAACCTGCCCAAGG
TCACAGGTGTTAGGTGGCAGAGCCAAGATGTTATTGTCAGTCTGACTTCAGGTGCATAAAACCTGAGGCTGAGCCTCCCC
ATGAGAGGAAAAATGTGTTTGTGGTGTGACCTCAGTTGCCAGGGAACTTGTGCAGGAAGCCGGGAGAGATGGCACAGA
CCCACCTGTGCTCACAGTACATGTTAAGAATTCCCAAGTCTCAGGCAAACCATCTCCAAGGGTGTTCACAGAAGACACACC
TTAAAGGCAAACGGGTTCATGATGTAATCTTCTATTGACTAGCTGTGAACCTCATTTTTCTTATCTGTGAAGTGGGAACAG
TAATAATCCAAGTCTTTTTATGGAGTTATCAAGATCAAAAAAGGAATGGATATACAAAGTGTTTTGTGAAATAAAAGCTCC
CTAAAATGGTAATTATTTTGTCCTCTGTTAGTGTCGGTAGTTATTGATATCTACATAGTACAGGTTATTTAAATATGTTTTAT
TCTGTAAAGATGGGCTGCCAAGTTTGTGTTCTGACCCTGTACGTGGTCCCTATGGTGTGATGAATGCCTGGGATCGCAGA
CTCCTGGATCTGTTCAGCTCCTGACTGGTCACAACCAGCCATGCTGGATGCATCATTCCCTCCCTGGGCCTCAGTGTGCCCT
CCTGCCAGGTGGTCACACATAAGACCTGTGCCACCTCTGCCCAGGGTTCCTATGAGAATCAATCGAGTTGATGAATGCAA
CCATGGCCTGAGAAAGACAAGACGCTTTAGCTATCAGATGGGCAGAAACAAGTATGTGTTGGTAAGAATATGGATTAAA
GGTTGGGAGGCCAAGATGGGTGGATCATGAGGTCAGAAGATTGAGACCACCCTGGATAACACGGTGAAACCCTGTCTCT
ACTAATAATACAAAAAATTAGCCGGGCATGGTGGCACGTGCCTGTAGTCCCAGCTACTCGGGAGGCTGAGGCAGGAGAA
TCACTTGGACCTGGGAGGTGGAGGTTGCAGTGAGCCAAGATTGCGCCACTGCACTCCAGCCTGGGTGACAGAGCGAGAC
TCCATCTCAAAAAAAACAAAACAAAAAAAATGTGGATTAAAGGAAACTTAGATATGCTGCCAGTGAGAAACTGTGCAAAG

CAATTTGGAAATAACCAGTAAAACTAAAGCTAAACGCATCCTTTGACCTAGCAGTTTTCCTCCTAGTCTTGGGAGGAACTC
TAGCACAGGGGCAAAAGGAAATGTACAAGAATGTTCACTGATGGCTTTGTTTGTATTTTTAAAAAGTTGAAAACAA

CCTAAATGATGATCAGCATAAGAACAAATAAATTGTGGTATTTTCCTAAAATTGAATATTACTCAACAGGTAAAAAAGAAT
GAACCAGAGCTATATGTATCAATATGGATAAATCTCAGAAACCTACTGTTGAGATGGAGGGTAGGGGAGTGAACTGCGG
AAGGATACCACTTATGTCATATTTCTAACTAAATGTATACGTTGATACTTTATTGTTTATGGCTACACACACATCTGGGTAA
TAGAGGTATAAAAGCCTGCACAAGAATGCTGTCAATATCACAATCAGTCATAGGGAGTGCTCCTGGGAGGGCCGCGGAG
AGGAGGGAACTAGGGATGCCACTGCAAGGGTAAGTAGGACGCTGGACTTCAACTTCAACTATACACATAACATTTCATTT
ATTTTTTTAAAATCCCCATCAAATGTAGAATGACAGGATTTTTCAGAGGTGGGTGATGTGTGCACAGATGTGGGCTATCTT
CTCCACACTCCTCTGTATATTTGATATTCTTTGCAATAAAAGAGAACAAGCCACAATacaaataggagg
